# Molecular Docking and Functional Analyses Reveal the Chondroprotective and Anti‐Inflammatory Potential of Statins in Osteoarthritis

**DOI:** 10.1111/jcmm.70791

**Published:** 2025-08-19

**Authors:** Bugrahan Regaip Kilinc, Feyza Kostak, Omer Faruk Yilmaz, Suray Pehlivanoglu, Duygu Yasar Sirin

**Affiliations:** ^1^ Department of Biology Institute of Natural and Applied Sciences, Namık Kemal University Tekirdag Turkey; ^2^ Department of Molecular Biology and Genetics Necmettin Erbakan University Konya Turkey; ^3^ Corlu State Hospital Tekirdag Turkey

**Keywords:** anti‐inflammatory effects, chemical‐protein interaction, chondroprotection, docking analyses, in silico experiments, pitavastatin, rosuvastatin

## Abstract

Osteoarthritis is a progressive degenerative joint disorder characterised by cartilage degradation and chronic inflammation. Beyond their well‐established lipid‐lowering properties, statins, particularly pitavastatin and rosuvastatin, have demonstrated promising anti‐inflammatory and chondroprotective effects. This study comprehensively evaluated these effects on human primary chondrocytes using an integrative approach involving in silico and in vitro experiments. Key molecular targets, including NF‐κB, IL‐1β and SOX9, were analysed to elucidate the mechanisms underlying the therapeutic potential of these statins. Molecular docking analyses using the CB‐Dock2 platform revealed strong binding affinities of both statins with the target proteins, with pitavastatin exhibiting a higher binding score (−8.0) compared to rosuvastatin (−7.9). Bioinformatics analyses via STITCH and STRING databases highlighted the involvement of both statins in pathways regulating inflammation, lipid metabolism, and cartilage homeostasis. In vitro experiments demonstrated that both statins preserved chondrocyte viability at 24 and 48 h; however, prolonged exposure led to significant declines, with rosuvastatin exhibiting greater cytotoxicity. Western blot analyses confirmed that both statins effectively suppressed IL‐1β expression, indicative of potent anti‐inflammatory activity. Pitavastatin transiently enhanced SOX9 expression, peaking at 24 h before declining, while rosuvastatin showed a more sustained but moderate increase. NF‐κB expression steadily increased over time with both statins, suggesting potential activation of compensatory inflammatory pathways during prolonged exposure. These findings underscore the dual anti‐inflammatory and chondroprotective roles of pitavastatin and rosuvastatin, while highlighting the need for careful consideration of dosage and treatment duration to mitigate cytotoxic effects and provide novel insights into the molecular mechanisms of statins.

**Abbreviations**
ANOVA: analysis of variance
AO/PI: acridine orange/propidium iodide
BMP‐2: bone morphogenetic protein 2
CAV1: caveolin‐1
Cavity Volume (Å^3^): binding pocket volume measured in cubic angstroms
CB‐Dock2: cavity detection‐based docking platform 2
CRP: C‐reactive protein
DMEM: Dulbecco's Modified Eagle Medium
DMSO: dimethyl sulfoxide
FBS: fetal bovine serum
HBSS: Hank's balanced salt solution
HIF‐1α: hypoxia‐inducible factor 1 alpha
HMG‐CoA: 3‐hydroxy‐3‐methylglutaryl‐CoA
HRP: horseradish peroxidase
IL‐1β: interleukin‐1 beta
MTT: 3‐(4,5‐dimethylthiazol‐2‐yl)‐2,5‐diphenyltetrazolium bromide
NF‐κB: nuclear factor kappa B.
NOS3: endothelial nitric oxide synthase
OA: osteoarthritis
OLR1: oxidised low‐density lipoprotein receptor 1
PBS: phosphate‐buffered saline
PCSK9: proprotein convertase subtilisin/kexin type 9
PDB: protein data bank
PON1: paraoxonase 1
PPI: protein–protein interaction
PVDF: polyvinylidene difluoride
RHOA: Ras homologue family member A
SD: standard deviation
SOX9: SRY‐box transcription factor 9
STITCH: search tool for interactions of chemicals
STRING: search tool for the retrieval of interacting genes/proteins
Tukey's Test: post hoc statistical test
Vina Score: docking score generated by AutoDock Vina

## 1 Introduction

Osteoarthritis (OA) is a chronic degenerative joint disease characterised by the progressive loss of articular cartilage, synovial inflammation, and subchondral bone remodelling, ultimately leading to joint pain and impaired mobility [1]. The disease process involves complex interactions between pro‐inflammatory cytokines, oxidative stress and mechanical stressors, with key molecular players such as NF‐κB, IL‐1β and SOX9 regulating inflammatory signalling and cartilage homeostasis [2, 3, 4, 5]. Despite its high prevalence and significant impact on quality of life, current treatments for OA remain largely palliative, focusing on symptom management rather than addressing the underlying pathophysiology [6].

Statins, inhibitors of 3‐hydroxy‐3‐methylglutaryl‐CoA reductase (HMG‐CoA reductase), are widely used for their lipid‐lowering effects in cardiovascular diseases [7]. Beyond their primary role, statins have been shown to exert pleiotropic effects, including anti‐inflammatory, antioxidant and immunomodulatory properties [8, 9]. These effects are mediated through their ability to modulate key signalling pathways, such as NF‐κB, which plays a central role in the inflammatory response [10, 11]. Emerging evidence suggests that statins may also influence cartilage‐specific pathways, including the regulation of SOX9, a transcription factor critical for chondrocyte differentiation and cartilage maintenance [12].

While previous studies have explored the general anti‐inflammatory properties of statins [13, 14, 15], their specific effects on human primary chondrocytes and cartilage metabolism remain poorly understood. Pitavastatin and rosuvastatin, two potent HMG‐CoA reductase inhibitors, have demonstrated distinct pharmacological profiles, making them promising candidates for further investigation in the context of cartilage health [16]. The current study hypothesises that pitavastatin and rosuvastatin exert anti‐inflammatory and chondroprotective effects on human primary chondrocytes by modulating key pathways involving NF‐κB, IL‐1β and SOX9; using a combination of computational, bioinformatics, and experimental approaches, this research aims to elucidate the molecular mechanisms underlying these effects, thereby contributing to the development of innovative strategies for managing OA and improving cartilage health.

## 2 Materials and Methods

### 2.1 Molecular Docking Analysis

Molecular docking analysis was performed using the CB‐Dock2 platform (http://cao.labshare.cn/cb‐dock/), which utilises cavity detection‐guided blind docking to predict binding sites and affinities. The three‐dimensional structures of the target proteins NF‐κB (PDB ID: 1NFI), SOX9 (PDB ID: 4EUW) and IL‐1β (PDB ID: 8RYS) were retrieved from the Protein Data Bank (http://www.rcsb.org). The 3D structures of the ligands pitavastatin (CID 5282452) and rosuvastatin (CID 446157) were obtained from the PubChem Compound database (https://pubchem.ncbi.nlm.nih.gov). The docking scores, binding poses and contact residues were analysed to evaluate the interaction strength and potential therapeutic relevance of the drugs with the target proteins [17].

### 2.2 Chemical‐Protein Interaction (STITCH) Analysis

The identification of proteins interacting with pitavastatin and rosuvastatin was conducted using the Search Tool for Interactions of Chemicals (STITCH) database, a comprehensive bioinformatics resource integrating experimentally validated, literature‐curated, and predicted interactions between chemical compounds and proteins. Interaction networks were constructed to elucidate the molecular mechanisms and pathways associated with the compounds. For this analysis, a confidence score threshold of ≥ 0.4 was applied to include interactions of moderate confidence; thereby, expanding the scope of potential protein targets. Proteins identified within the network were considered significant based on their interactions with the compounds. The resulting interaction networks were subsequently exported to the STRING (Search Tool for the Retrieval of Interacting Genes/Proteins) database for further bioinformatic analysis and functional characterisation [18].

### 2.3 Protein–Protein Interaction (PPI) Analyses

Protein–protein interaction networks were constructed to explore the broader interaction landscape of the identified genes, including NF‐κB, SOX9 and IL‐1β, in the context of HMG‐CoA reductase inhibitor treatment. The STRING database was utilised for the prediction and visualisation of interaction networks. To ensure the robustness of the analysis, a confidence score threshold of > 0.4 was applied. Key hub proteins within the network were identified through degree centrality analysis, highlighting nodes with significant connectivity. Functional enrichment analysis was performed to uncover the biological processes, molecular functions, and pathways associated with these interactions. This approach provided insights into the molecular mechanisms potentially regulated by pitavastatin and rosuvastatin treatment [19].

### 2.4 Preparation of Human Primary Chondrocyte Cultures

Human primary chondrocyte cultures were prepared from cartilage tissue obtained from patients undergoing total knee arthroplasty (four males, four females; mean age: 44.12 ± 4.87 years) which are classified as Kellgren–Lawrence radiographic Grade IV. The tissues were transported aseptically in sterile Falcon tubes containing Dulbecco's Modified Eagle Medium (DMEM; Cat#11965092; Thermo Fisher Scientific) with 5% penicillin–streptomycin (Cat#15140122; Thermo Fisher Scientific). After washing with phosphate‐buffered saline (PBS), tissues were mechanically dissociated and digested overnight using Clostridium histolyticum collagenase type I (475 μg/mL) and type II (125 μg/mL) (Cat#J13820.03 and Cat#17101015; Thermo Fisher Scientific) in Hank's Balanced Salt Solution (HBSS; Cat#14170070; Thermo Fisher Scientific) at 37.4°C and 5% CO_2_. The cells were centrifuged at 1300 rpm for 10 min; resuspended in DMEM with 10% fetal bovine serum (FBS; Cat# A5670801; Thermo Fisher Scientific), and incubated at 37°C with 5% CO_2_. Primary chondrocytes were used for experiments after 21 days of culture [20].

### 2.5 Application of Pitavastatin and Rosuvastatin to Cultures

Pitavastatin (1 μM) and rosuvastatin (20 μM) were dissolved in DMEM and applied to the primary chondrocyte cultures. The control group received only culture medium without statins. Treatments were applied for 24, 48 and 72 h, and all experiments were performed in triplicate.

### 2.6 MTT Assay for Cell Viability and Proliferation

Cell viability was assessed using the MTT (Vybrant MTT Cell Proliferation Assay Kit, V‐13154; Invitrogen) assay. Cells were seeded in 96‐well plates at a density of 1.5 × 10^6^ cells per well. After treatment, 100 μL of MTT solution (12 mM) was added to each well and incubated for 2 h. Formazan crystals were dissolved using dimethyl sulfoxide (DMSO), and absorbance was measured at 540 nm. The viability of control cells was set to 100%, and cell viability in treated groups was calculated accordingly. Half maximal inhibitory concentrations (IC50) were tested by MTT assay to detect cellular sensitivity to pitavastatin and rosuvastatin.

### 2.7 AO/PI Staining

Acridine Orange (AO) and Propidium Iodide (PI) staining were used to assess cell viability after 24, 48 and 72 h of treatment. A mixture of 4 mg/mL AO and 4 mg/mL PI was used to stain the cells. Viable cells emitted green fluorescence, and apoptotic or necrotic cells emitted red fluorescence, observed under a fluorescence microscope (Leica, DM 2500).

### 2.8 Western Blotting for SOX9, NF‐κB and IL‐1β

Proteins from treated and control cells were extracted using RIPA buffer and quantified using the Bradford assay. Equal amounts of protein were separated by 10% SDS‐PAGE and transferred to polyvinylidene difluoride (PVDF) membranes. Membranes were blocked with 5% non‐fat milk and incubated overnight with primary antibodies against SOX9 (Cat#MA5‐17177; Thermo Fisher Scientific), NF‐κB (Cat#MA5‐15563; Thermo Fisher Scientific), IL‐1β (Cat#MA5‐23691; Thermo Fisher Scientific) and β‐actin (Cat#MA1‐140; Thermo Fisher Scientific). After washing, membranes were incubated with WesternBreeze Chemiluminescent Kit (Cat# WB7104; Thermo Fisher Scientific) containing HRP‐conjugated secondary antibodies, and protein bands were visualised after exposure to chemiluminescent sensitive X‐ray films. ImageJ software was used to analyse band intensities, and β‐actin served as a loading control [12].

### 2.9 Statistical Analysis

All experiments were performed in triplicate. Data were expressed as mean ± standard deviation (SD). Statistical comparisons were made using one‐way analysis of variance (ANOVA) followed by Tukey's post hoc test. A *p*‐value of < 0.05 was considered statistically significant.

## 3 Result

This study explored the effects of pitavastatin and rosuvastatin on human primary chondrocytes using a multi‐faceted approach that combined in silico molecular docking, bioinformatics analyses, and in vitro experimental evaluations. The molecular docking analysis provided detailed insights into the binding affinities, cavity volumes, interaction sites, and contact residues of both statins with key target proteins, including IL‐1β, SOX9 and NF‐κB, emphasising their potential anti‐inflammatory and chondroprotective properties. Bioinformatics analyses were employed to further elucidate the broader interaction landscape of these compounds. Chemical‐protein interaction studies using the STITCH database identified key protein targets and constructed interaction networks to highlight potential pathways modulated by pitavastatin and rosuvastatin. Additionally, PPI network analyses provided a deeper understanding of the functional relationships between identified targets, enabling the identification of critical hub proteins and enriched biological pathways associated with statin‐mediated effects. Complementing the in silico findings, in vitro experiments evaluated cell viability, protein expression levels, and morphological changes in human primary chondrocytes. These experiments validated computational predictions and revealed the time‐dependent effects of pitavastatin and rosuvastatin on cartilage‐specific pathways and inflammatory processes. Together, the integration of in silico and bioinformatics approaches with in vitro validation offers a comprehensive understanding of the mechanisms underlying the therapeutic potential of these statins. Below, the results of molecular docking, bioinformatics analyses, and in vitro studies are presented in detail.

**FIGURE 1:**
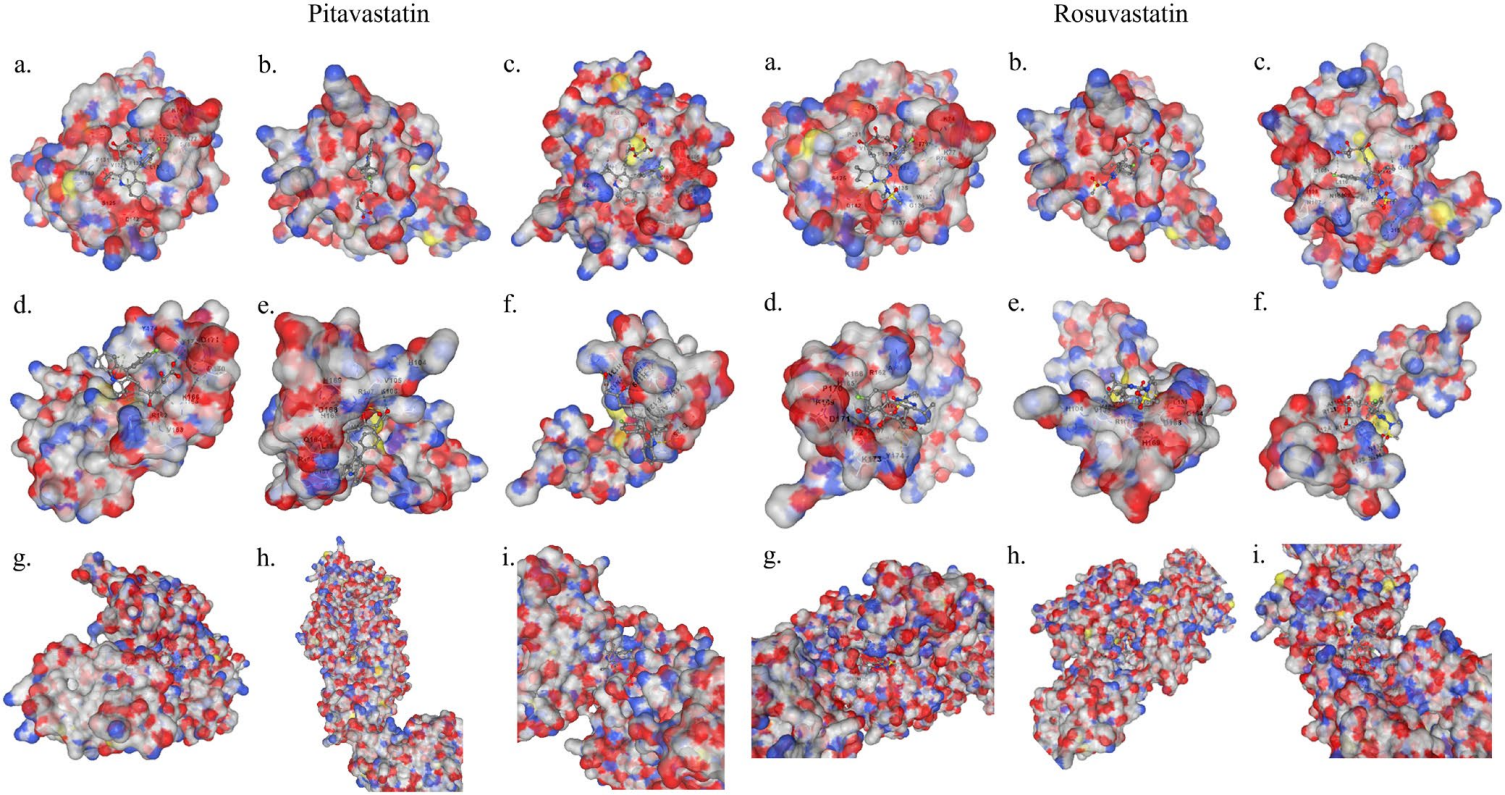
Docking results of the genes NF‐κB, SOX9 and IL‐1β with ligands pitavastatin and rosuvastatin using the CB‐Dock2 platform.

Figure 1 illustrates the docking study results for all drugs and proteins analysed. Table 1 provides a detailed summary of the docking parameters, including the highest Vina score, cavity volume (Å^3^), centre coordinates (*x*, *y*, *z*), docking box size (*x*, *y*, *z*), and contact residues involved in the binding (Figure 1). The docking scores, binding poses, and contact residues were analysed to evaluate the interaction strength and therapeutic potential of pitavastatin and rosuvastatin with the target proteins (Table 1).

**TABLE 1:** Molecular docking analysis results of pitavastatin and rosuvastatin with IL‐1β, SOX9 and NF‐κB.

Ligand	Receptor	Highest Vina score	Cavity volume (Å^3^)	Centre (*x*, *y*, *z*)	Docking size (*x*, *y*, *z*)	Contact residues
Pitavastatin	IL‐1β	1: −7.4	133	8, −23, 6	22, 22, 22	Chain A: SER21 GLY22 PRO23 TYR24 GLU25 LYS74 ASP75 LYS77 PRO78 THR79 LEU80 GLN81 LEU82 GLU83 SER84 TRP120 SER125 MET130 PRO131 VAL132 PHE133 LEU134 GLY135 GLY136 THR137 ASP142
		2: −6.9	199	13, −19, −14	22, 22, 22	Chain A: PRO2 VAL3 ARG4 SER5 LEU6 ASN7 PHE42 SER43 MET44 SER45 GLY61 LEU62 LYS63 GLU64 LYS65 ASN66 LEU67 TYR68 VAL85 PRO87 LYS88 ASN89 TYR90 PRO91 LYS92 VAL151 SER152 SER153
		3: −6.6	91	15, −1, −3	22, 22, 22	Chain A: ARG4 LEU6 ARG11 MET44 PHE46 GLU51 SER52 ASN53 ASP54 ILE56 LYS103 GLU105 ASN107 ASN108 LYS109 LEU110 PHE146 THR147 MET148 GLN149 PHE150
	SOX9	1: −7.0	347	−4, −19, 16	22, 22, 22	Chain A: ARG107 PRO108 MET109 ASN110 ALA111 PHE112 MET113 TRP143 LYS151 VAL155 ALA158 GLU159 LEU161 ARG162 VAL163 HIS165 LYS166 LYS167 HIS169 PRO170 ASP171 TYR172 LYS173 TYR174
		2: −6.2	180	−8, −27, 9	22, 22, 22	Chain A: HIS104 VAL105 LYS106 ARG107 PRO108 MET109 GLN117 GLU157 ARG160 LEU161 GLN164 HIS165 ASP168 HIS169
		2: −5.3	514	−28, −23, 11	22, 22, 22	Chain A: MET109 PHE112 MET113 ALA116 GLN117 ALA118 ARG120 ARG121 ALA124 ASP125 TYR127 PRO128 HIS129 LEU130 HIS131 ASN132 ALA133 LEU135 SER136
	NF‐κB	1: −8.0	904	−11, 53, −11	22, 22, 22	Chain A: ASN200 ARG201 SER205 GLY208 GLY209 ASP210 GLU211 ILE212 PHE213 ARG253 ASP291 Chain B: ARG255 ASP257 ARG258 Chain F: ASP141 ARG143 GLY144 ASN145 GLU153 ASN180 TYR181 ASN182 GLY183 HIS184 LEU189 ILE192 HIS193 GLU213 CYS215 ARG218 LEU223 ASP226 LEU227
		2: −7.8	798	−2, 78, 85	22, 22, 22	Chain C: ASN200 ARG201 SER205 LEU207 GLY208 GLY209 ASP210 GLU211 ILE212 PHE213 ARG253 ASP291 Chain D: ARG255 ASP257 ARG258 Chain E: ARG143 ASN145 GLU153 ASN180 ASN182 HIS184 LEU189 ILE192 HIS193 GLU213 CYS215 ARG218 LEU223 ASP226 LEU227
		3: −7.6	3002	−7, 51, 11	31, 22, 22	Chain A: LYS28 GLN29 ARG30 GLU49 ARG50 SER51 THR52 LYS79 ASP80 ARG158 HIS181 PRO182 PHE184 LYS218 VAL219 GLN220 LYS221 GLU222 ASP223 ILE224 GLU225 VAL226 SER240 GLN241 ALA242 VAL244 HIS245 ARG246 GLN247 PRO275 Chain B: LYS252 VAL254 ASP274 Chain F: GLN249 GLY250 TYR251 THR257 TRP258 GLY259 ARG260 MET279 LEU280 PRO281 GLU282
Rosuvastatin	IL‐1β	1: −7.2	133	8, −23, 6	22, 22, 22	Chain A: SER21 GLY22 PRO23 TYR24 GLU25 LEU26 LEU69 LYS74 LYS77 PRO78 THR79 LEU80 GLN81 LEU82 GLU83 SER84 TRP120 SER123 SER125 MET130 PRO131 VAL132 PHE133 LEU134 GLY135 GLY136 THR137 ASP142
		2: −6.4	199	13, −19, −14	22, 22, 22	Chain A: PRO2 VAL3 ARG4 SER5 LEU6 ASN7 SER43 MET44 SER45 GLY61 LEU62 LYS63 GLU64 LYS65 ASN66 LEU67 TYR68 VAL85 ASP86 PRO87 LYS88 ASN89 TYR90 PRO91 LYS92 SER153
		3: −5.9	91	15, −1, −3	22, 22, 22	Chain A: LEU6 ARG11 GLN15 MET44 PHE46 ASN53 ILE56 LYS103 GLU105 ILE106 ASN107 ASN108 LYS109 LEU110 PHE146 THR147 MET148 GLN149 PHE150
	SOX9	1: −5.8	347	−4, −19, 16	22, 22, 22	Chain A: ARG107 PRO108 MET109 ASN110 ALA111 PHE112 MET113 TRP143 LYS151 VAL155 GLU156 ALA158 GLU159 ARG162 VAL163 HIS165 LYS166 LYS167 HIS169 PRO170 ASP171 TYR172 LYS173 TYR174
		2: −5.5	180	−8, −27, 9	22, 22, 22	Chain A: HIS104 VAL105 LYS106 ARG107 PRO108 MET109 VAL114 GLN117 GLU157 ARG160 LEU161 ARG162 GLN164 HIS165 ASP168 HIS169
		3: −5.2	514	−28, −23, 11	22, 22, 22	Chain A: MET109 PHE112 MET113 ALA116 GLN117 ALA118 ARG120 ARG121 ALA124 ASP125 GLN126 TYR127 PRO128 HIS129 LEU130 HIS131 ASN132 ALA133 LEU135 SER136
	NF‐κB	1: −7.9	3597	−3, 79, 106	32, 22, 22	Chain C: LYS28 ARG30 GLY31 GLU49 ARG50 SER51 THR52 VAL219 GLN220 LYS221 GLU222 ASP223 ILE224 GLU225 SER238 PHE239 SER240 GLN241 ALA242 VAL244 HIS245 ARG246 GLN247 PRO275 SER276 Chain D: ASN250 LYS252 VAL254 ASP274 Chain E: TYR248 GLN249 GLY250 TYR251 THR257 TRP258 GLY259 ARG260 PRO261 SER262 GLN266 MET279 LEU280 PRO281 GLU282
		2: −7.3	904	−11, 53, −11	22, 22, 22	Chain A: ASN200 ARG201 GLY208 GLY209 ASP210 GLU211 ILE212 PHE213 ARG253 Chain B: ARG255 MET256 ASP257 ARG258 Chain F: ARG143 ASN145 LEU150 GLU153 ASN180 TYR181 ASN182 GLY183 HIS184 LEU189 ILE192 HIS193 GLU213 CYS215 ASN216 ARG218 LEU223 ASP226 LEU227
		3: −7.0	3002	−7, 51, 11	31, 22, 22	Chain A: LYS28 GLN29 ARG30 GLY31 PRO47 GLU49 ARG50 SER51 THR52 ASP53 THR54 LYS79 ASP80 ARG158 HIS181 PRO182 PHE184 LYS218 VAL219 GLN220 LYS221 GLU222 ASP223 ILE224 GLU225 VAL226 ARG236 GLY237 SER238 PHE239 SER240 GLN241 ALA242 VAL244 HIS245 ARG246 GLN247 ARG274 PRO275 SER276 Chain F: THR257 TRP258 GLY259 ARG260 PRO261 SER262 THR263 GLN266 MET279 LEU280 PRO281 GLU282

Among the proteins analysed, NF‐κB displayed the highest binding affinities with both ligands, suggesting its central role as a target for the therapeutic effects of these statins. Pitavastatin exhibited a particularly strong interaction with NF‐κB, achieving the highest Vina score (−8.0) and occupying a large cavity volume (904 Å^3^). Key contact residues such as ARG201, GLU211 and LEU223 were identified, implicating regions critical for NF‐κB's biological function, such as DNA binding and dimerisation domains [21]. Rosuvastatin similarly demonstrated a robust interaction with NF‐κB, with a Vina score of −7.9 and interactions mediated by residues such as LYS28, SER240 and HIS245 [22].

The analysis of interactions with IL‐1β highlighted its susceptibility to modulation by both statins. Pitavastatin exhibited the highest binding affinity (−7.4) within a cavity volume of 133 Å^3^, engaging key residues such as SER21, TYR24 and GLU25. These residues are known to play important roles in IL‐1β‐mediated inflammatory signalling. Rosuvastatin showed a slightly lower binding affinity (−7.2) but interacted with overlapping residues, including TYR24 and GLU25, indicating similar binding mechanisms. These results suggest that both statins may effectively target IL‐1β to mitigate inflammation in cartilage‐related disorders.

For SOX9, a critical transcription factor involved in cartilage homeostasis, pitavastatin demonstrated a higher binding affinity (−7.0) compared to rosuvastatin (−5.8). Pitavastatin interacted with residues such as ARG107, MET109 and LYS151, which are associated with the protein's regulatory domains. Rosuvastatin, while showing a lower binding affinity, also targeted essential residues, including ARG107 and GLU159. The differential binding patterns observed for SOX9 suggest that pitavastatin may exert stronger chondroprotective effects by enhancing cartilage‐specific transcriptional pathways (Table 1).

The combined analysis of STITCH and PPI data revealed key protein interactions and pathways associated with pitavastatin and rosuvastatin, highlighting their potential anti‐inflammatory and chondroprotective effects. Both statins demonstrated interactions with HMGCR (3‐hydroxy‐3‐methylglutaryl‐CoA reductase), confirming their primary role in inhibiting cholesterol biosynthesis [23]. Additionally, interactions with NOS3 (endothelial nitric oxide synthase) and RHOA (Ras homologue family member A) suggest broader roles in cellular signalling and nitric oxide production, which may contribute to anti‐inflammatory effects [24].

For pitavastatin, STITCH results emphasised its connections with proteins like PON1 (paraoxonase 1), which is associated with antioxidant defence [25] and OLR1 (oxidised low‐density lipoprotein receptor 1), implicating a role in modulating oxidative stress [26]. The PPI analysis further highlighted its interactions with NF‐κB1, NF‐κB2 and IL‐1β, suggesting that pitavastatin may attenuate inflammatory pathways by modulating NF‐κB activation and cytokine production [27]. The inclusion of SOX9, a transcription factor critical for cartilage maintenance, underscores pitavastatin's potential chondroprotective properties.

Rosuvastatin exhibited similar molecular interactions, with notable connections to CRP (C‐reactive protein) and PCSK9 (proprotein convertase subtilisin/kexin type 9), reflecting its roles in systemic inflammation regulation and lipid metabolism [28]. The PPI analysis reinforced its impact on inflammation through strong interactions with NF‐κB1, NF‐κB2 and IL‐1β, while also highlighting its potential effect on cartilage metabolism via SOX9. The interaction with CAV1 (caveolin‐1) suggests additional roles in cellular signalling and membrane organisation [29, 30].

**FIGURE 2:**
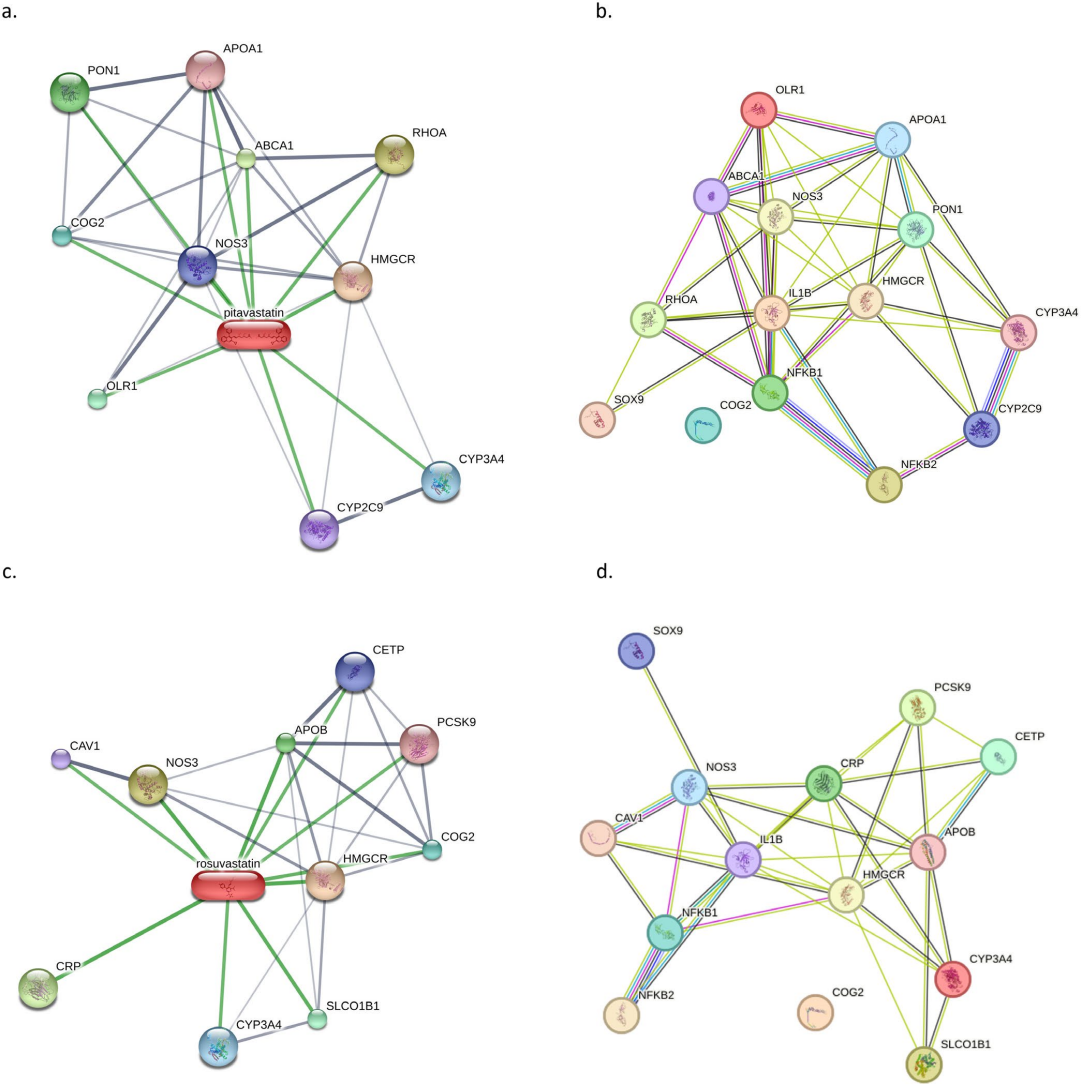
Interaction network analysis of NFKB1, NFKB2, SOX9, IL1B, HMGCR, and their interacting proteins in human primary chondrocytes treated with pitavastatin and rosuvastatin. The PPI networks were divided into two categories: (a, b) Pitavastatin‐mediated chemical‐protein interaction network and the associated target genes' PPI network; (c, d) Rosuvastatin‐mediated chemical‐protein interaction network and the associated target genes' PPI network.

The functional enrichment of these networks identified key biological processes, including the regulation of inflammation, cartilage‐specific pathways and lipid metabolism. Both statins demonstrated robust interaction networks that align with their hypothesised effects on reducing inflammation and enhancing cartilage health. These results support their potential therapeutic applications beyond lipid‐lowering, particularly in inflammatory and chondroprotective contexts (Figure 2).

**FIGURE 3:**
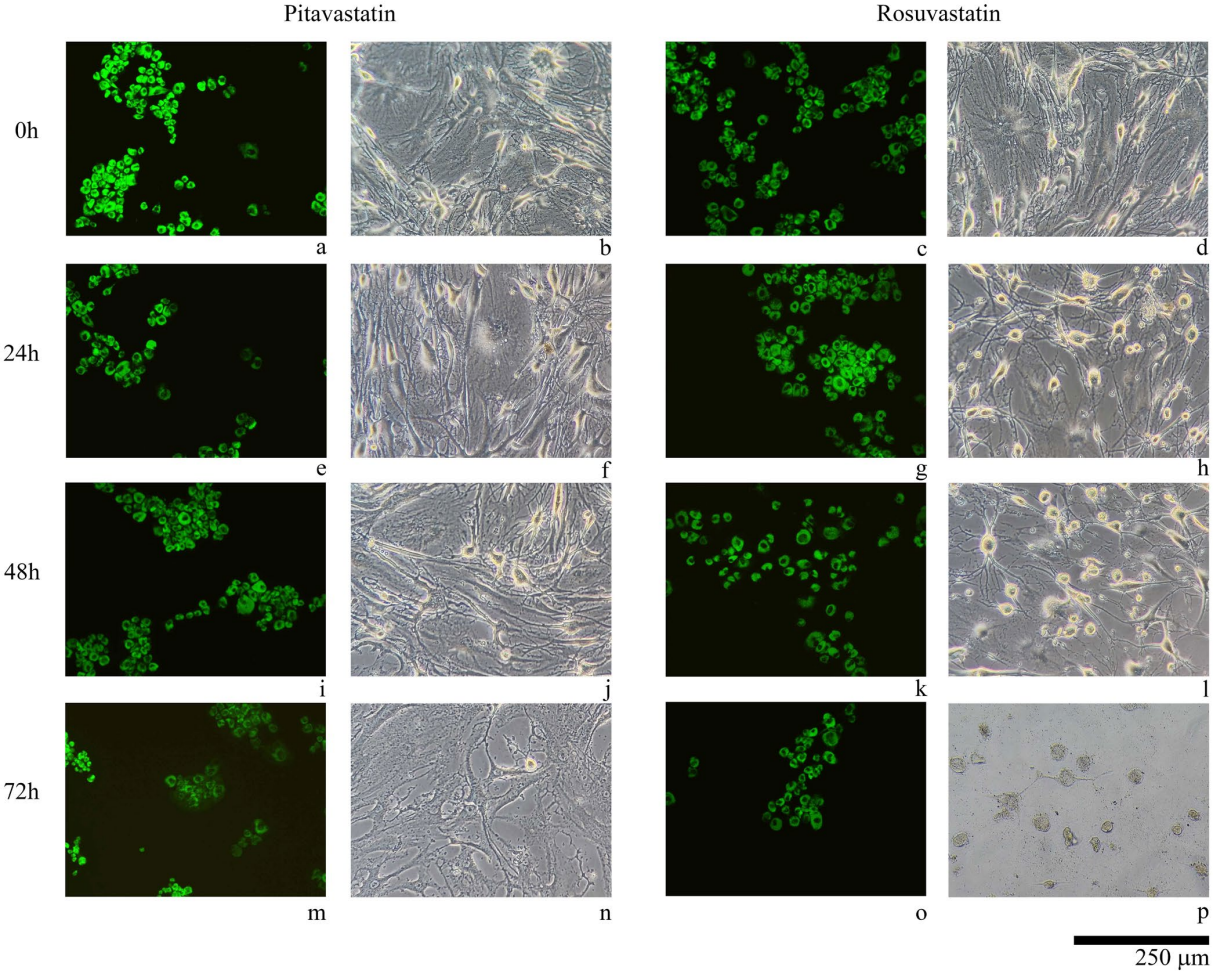
Microscopic evaluation of chondrocyte cultures. First Column: Acridine orange/propidium iodide‐stained cultures (10× magnification), Second Column: Inverted microscopy images (40× magnification). First Lane (a–d) Control Group Samples, (e, f) Micrographs of Pitavastatin‐applied cultures for 24 h, (g, h) Micrographs of Rosuvastatin‐applied cultures for 24 h, (i, j): Micrographs of Pitavastatin‐applied cultures for 48 h, (k, l) Micrographs of Rosuvastatin‐applied cultures for 48 h, (m, n) Micrographs of Pitavastatin‐applied cultures for 72 h, (o, p) Micrographs of Rosuvastatin‐applied cultures for 72 h.

The effects of pitavastatin and rosuvastatin on human primary chondrocytes were assessed over 24, 48 and 72 h using AO/PI staining and inverted microscopy. In the control group, cell viability remained high throughout the experiment, with bright green fluorescence observed in AO/PI staining and intact cell morphology and extracellular matrix integrity under inverted microscopy. Pitavastatin‐treated cultures demonstrated preserved cell viability and matrix integrity at 24 and 48 h, with predominantly green fluorescence and healthy cell morphology. However, at 72 h, a noticeable decline in cell viability was observed, with a reduction in cell density and visible damage to the extracellular matrix, although no red fluorescence indicative of cell death was detected. Similarly, rosuvastatin‐treated cultures exhibited a gradual decline in cell viability over time. At 24 and 48 h, green fluorescence remained evident, but cell morphology showed minor structural alterations. By 72 h, significant structural damage and cell shrinkage were observed under microscopy, with a substantial reduction in cell density and matrix integrity, although no red fluorescence was detected (Figure 3). These results suggest that while both statins may provide short‐term chondroprotective effects, prolonged exposure results in adverse outcomes, with rosuvastatin causing more pronounced morphological damage and matrix degradation at later stages [31].

**FIGURE 4:**
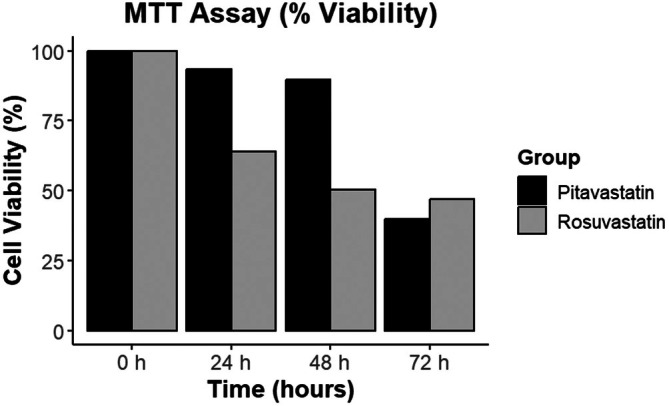
MTT assay showing the percentage cell viability of human primary chondrocytes treated with Pitavastatin and Rosuvastatin at different time points (Control, 0, 24, 48 and 72 h).

The MTT assay results, depicted in Figure 3, indicate the percentage cell viability of human primary chondrocytes treated with pitavastatin and rosuvastatin over various time points. At 0 h, both groups exhibited 100% viability, serving as a baseline. At 24 and 48 h, pitavastatin‐treated cells maintained higher viability compared to rosuvastatin‐treated cells, suggesting its superior short‐term protective effects on chondrocytes. However, by 72 h, both groups demonstrated a significant decline in viability, with rosuvastatin showing a more pronounced reduction (Figure 4). This indicates that while both statins support chondrocyte viability in the short term, prolonged exposure adversely affects cell survival, with rosuvastatin having a more detrimental impact [31].

For pitavastatin‐treated cells, IL‐1β expression progressively decreased over time, indicating a notable anti‐inflammatory effect [32]. NF‐κB expression, however, showed a gradual increase, suggesting an activation of inflammatory signalling pathways despite the reduction in IL‐1β levels. SOX9 expression peaked at 24 h, reflecting enhanced chondroprotective activity during early treatment, but declined at 48 h, indicating a reduction in cartilage maintenance signalling over time [12].

For rosuvastatin‐treated cells, IL‐1β levels also decreased over time, similar to pitavastatin, confirming its anti‐inflammatory properties [33]. However, NF‐κB expression showed a gradual increase, suggesting a concurrent activation of inflammatory pathways. SOX9 expression increased at 24 h and continued to rise slightly at 48 h, indicating a more sustained but moderate chondroprotective effect compared to pitavastatin.

**FIGURE 5:**
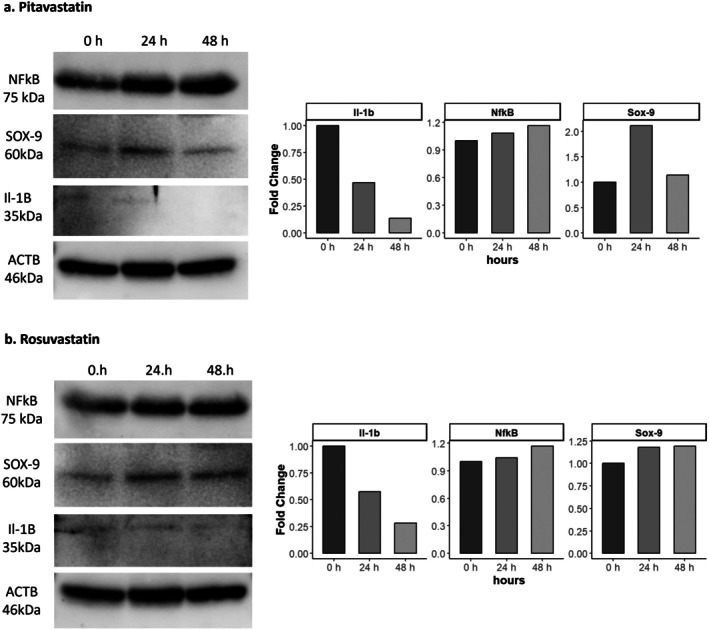
Western blot analysis of NFκB, Sox‐9, IL‐1β and βActin protein expression levels in human primary chondrocytes treated with (a) Pitavastatin and (b) Rosuvastatin across different time points (0, 24, 48 and 72 h).

These findings highlight that while both statins exhibit anti‐inflammatory and chondroprotective effects, their mechanisms and durations of action differ. Pitavastatin demonstrated a stronger but transient impact on SOX9 expression and a steady reduction in IL‐1β levels, accompanied by an increase in NF‐κB expression. In contrast, rosuvastatin showed a more sustained increase in SOX9 expression and a comparable reduction in IL‐1β, but with a gradual rise in NF‐κB levels, suggesting differential regulatory effects on inflammation and cartilage‐specific pathways (Figure 5). Comprehensive results from molecular docking analyses, expanded bioinformatics interaction networks, and the original uncropped Western blot images from our in vitro experiments are provided in Appendix S1.

## 4 Discussion

This study explored the anti‐inflammatory and chondroprotective effects of pitavastatin and rosuvastatin on human primary chondrocytes, providing valuable insights into their potential therapeutic applications in cartilage‐related disorders, including osteoarthritis. By integrating molecular docking, bioinformatics analyses, and in vitro experiments, the study revealed distinct yet complementary roles for these statins in modulating inflammatory and cartilage‐specific pathways.

Molecular docking analyses demonstrated strong binding affinities of both statins with NF‐κB, IL‐1β and SOX9, underscoring their dual role in regulating inflammation and maintaining cartilage homeostasis. Notably, pitavastatin exhibited a slightly higher binding affinity across all target proteins compared to rosuvastatin, particularly with NF‐κB, achieving the highest Vina score (−8.0). This suggests a more robust interaction with pathways governing inflammatory signalling and transcriptional regulation. Rosuvastatin, while displaying slightly lower affinities, showed consistent interactions with key residues, reinforcing its potential to modulate similar molecular pathways. These findings align with the pleiotropic properties of statins and their ability to act on multiple cellular targets beyond lipid‐lowering.

The bioinformatics analyses using STITCH and PPI networks provided further insights into the broader interaction landscape. Both statins demonstrated significant connectivity with proteins involved in lipid metabolism, oxidative stress regulation, and inflammatory signalling, including NF‐κB and IL‐1β. The identification of SOX9 as a critical hub protein highlights the role of these statins in cartilage‐specific pathways. While pitavastatin exhibited stronger interactions with antioxidant‐related proteins such as PON1, rosuvastatin showed notable connections with CRP and PCSK9, reflecting its role in systemic inflammation and lipid regulation. These differences emphasise the unique pharmacological profiles of pitavastatin and rosuvastatin, suggesting that they may exert complementary effects in clinical applications.

The in vitro findings corroborated the computational predictions, demonstrating time‐dependent effects of both statins on chondrocyte viability and protein expression. Cell viability assays revealed that both statins maintained viability at 24 and 48 h, with pitavastatin showing slightly superior protective effects. However, by 72 h, significant declines in viability were observed, particularly for rosuvastatin, suggesting potential cytotoxicity with prolonged exposure. These results highlight the importance of optimising dosage and exposure duration in potential therapeutic settings.

Western blot analyses provided mechanistic insights into the observed effects. Pitavastatin significantly reduced IL‐1β expression, indicating a strong anti‐inflammatory effect, while SOX9 expression peaked at 24 h, reflecting enhanced chondroprotective activity. However, the concurrent increase in NF‐κB expression at later time points suggests the activation of compensatory inflammatory pathways, which may limit its long‐term efficacy. Rosuvastatin similarly reduced IL‐1β levels and demonstrated a sustained but moderate increase in SOX9 expression, suggesting prolonged chondroprotective effects. Nevertheless, the gradual rise in NF‐κB expression underscores the potential risks associated with extended use, including the activation of pro‐inflammatory signalling.

These findings align with existing literature demonstrating the pleiotropic effects of statins, including their anti‐inflammatory and antioxidant properties. The differential effects observed between pitavastatin and rosuvastatin further highlight their distinct pharmacokinetic and pharmacodynamic profiles. While pitavastatin appears to exert stronger early effects on cartilage maintenance, rosuvastatin's sustained activity on SOX9 suggests potential advantages for prolonged use, albeit with careful monitoring due to its cytotoxicity at later time points.

One limitation of this study is its reliance on in vitro models, which do not fully replicate the complexity of the in vivo cartilage microenvironment or the systemic inflammatory milieu present in osteoarthritis. Future studies using animal models or clinical trials are needed to validate these findings and better understand the translational potential of statins in cartilage‐related disorders. Additionally, exploring downstream pathways such as oxidative stress markers, apoptosis regulators, and autophagy‐related proteins could provide a more comprehensive understanding of the molecular mechanisms underlying the observed effects.

In conclusion, this study demonstrates that pitavastatin and rosuvastatin exhibit significant anti‐inflammatory and chondroprotective properties by modulating key molecular pathways, including NF‐κB, IL‐1β and SOX9. However, the potential cytotoxicity observed with prolonged exposure underscores the need for careful consideration in therapeutic applications. These findings provide a strong foundation for further research into the use of statins as adjunct therapies for osteoarthritis and other cartilage‐related disorders.

## Author Contributions

**Bugrahan Regaip Kilinc:** writing – original draft (equal). **Feyza Kostak:** writing – original draft (equal). **Omer Faruk Yilmaz:** writing – original draft (equal). **Suray Pehlivanoglu:** writing – original draft (equal). **Duygu Yasar Sirin:** writing – original draft (equal).

## Ethics Statement

This study was conducted in accordance with the ethical standards of the (TNKÜ Girişimsel Olmayan Klinik Araştırmalar Etik Kurulu) and was approved under Ethics Approval Number: (2024.303.11.10).

## Consent

Written informed consent was obtained from all donors prior to cartilage tissue collection.

## Conflicts of Interest

The authors declare no conflicts of interest.

## Supporting information


**Appendix S1:** jcmm70791‐sup‐0001‐AppendixS1.pdf.

## Data Availability

The datasets generated and analysed during this study are available from the corresponding author upon reasonable request.
