# Supplementary material for: Molecular Docking and Functional Analyses Reveal the Chondroprotective and Anti‐Inflammatory Potential of Statins in Osteoarthritis
Source: J Cell Mol Med. 2025 Aug 19;29(16):e70791. doi: 10.1111/jcmm.70791 (PMC12364615; doi:10.1111/jcmm.70791)
Supplement: Supplementary file 1 — Appendix S1: jcmm70791‐sup‐0001‐AppendixS1.pdf. [file JCMM-29-e70791-s001.pdf]

Supplementary Information

This document contains raw data, extended analyses, and additional visualizations to support the main text of our manuscript, "MOLECULAR DOCKING AND FUNCTIONAL ANALYSES REVEAL THE CHONDROPROTECTIVE AND ANTI-INFLAMMATORY POTENTIAL OF STATINS IN OSTEOARTHRITIS". Our goal is to ensure the transparency and reproducibility of our findings. These supplementary materials include comprehensive results from molecular docking analyses, expanded bioinformatics interaction networks, and the original uncropped Western blot images from our in vitro experiments.

Supplementary Figures

Expanded Bioinformatics Analysis Networks

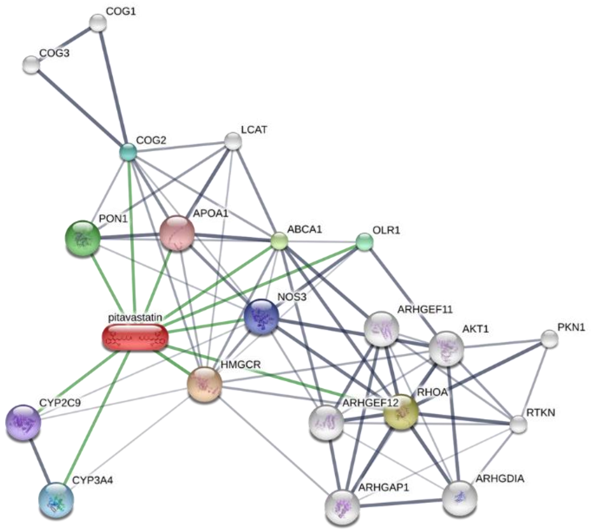

Figure S1: Pitavastatin Chemical-Protein Interaction Network This figure presents the direct chemical-protein interaction network for **Pitavastatin**, as constructed using the STITCH database. The network includes all interactions with a confidence score greater than 0.4, showing the primary protein targets of the drug.

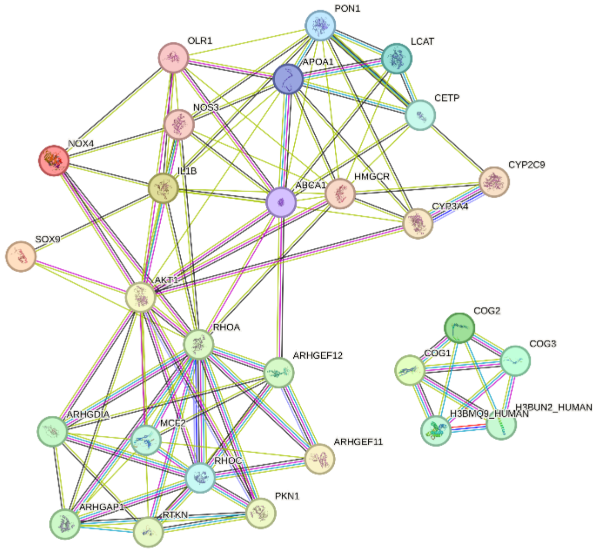

Figure S2: Pitavastatin-Associated Protein-Protein Interaction (PPI) Network This figure displays the protein-protein interaction (PPI) network of the proteins identified in Figure S1, along with the key targets of our study: **NF-κB**, **SOX9**, and **IL-1β**. Constructed using the STRING database, this expanded network highlights the functional relationships and potential pathways modulated by Pitavastatin.

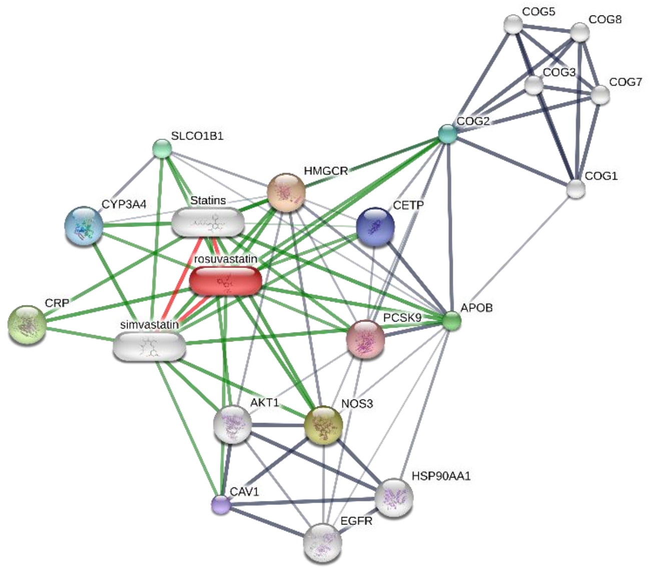

Figure S3: Rosuvastatin Chemical-Protein Interaction Network This figure presents the direct chemical-protein interaction network for **Rosuvastatin**, as constructed using the STITCH database. The network includes all interactions with a confidence score greater than 0.4, showing the primary protein targets of the drug.

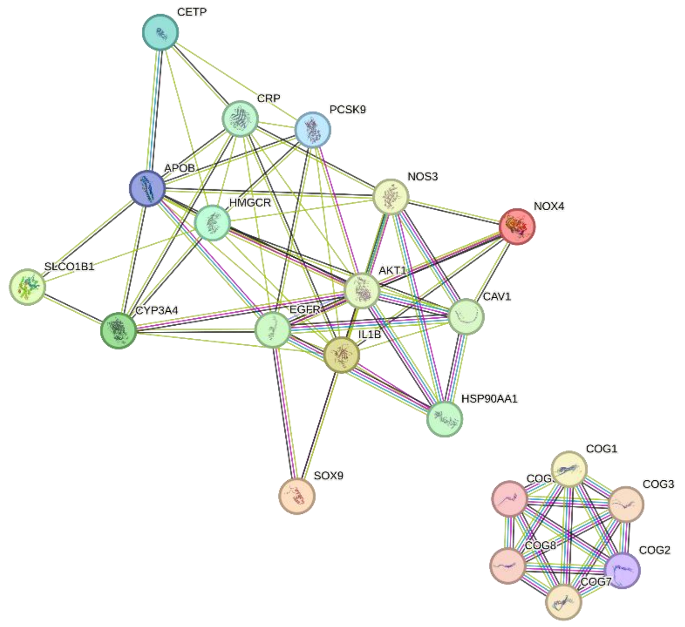

Figure S4: Rosuvastatin-Associated Protein-Protein Interaction (PPI) Network This figure displays the protein-protein interaction (PPI) network of the proteins identified in Figure S3, along with the key targets of our study: **NF-κB**, **SOX9**, and **IL-1β**. Constructed using the STRING database, this expanded network highlights the functional relationships and potential pathways modulated by Rosuvastatin.

Raw Western Blot Images

Figure S5: Raw Western Blot Image of Pitavastatin(Alipza) and Rosuvastatin -Treated Chondrocytes

This figure shows the original, uncropped raw Western blot film for Pitavastatin and Rosuvastatin treatment across all time points (0, 24, 48, 72 hours) in human primary chondrocytes. The image clearly displays the protein bands for SOX9, NF-κB, IL-1β, and the loading control, β-actin.

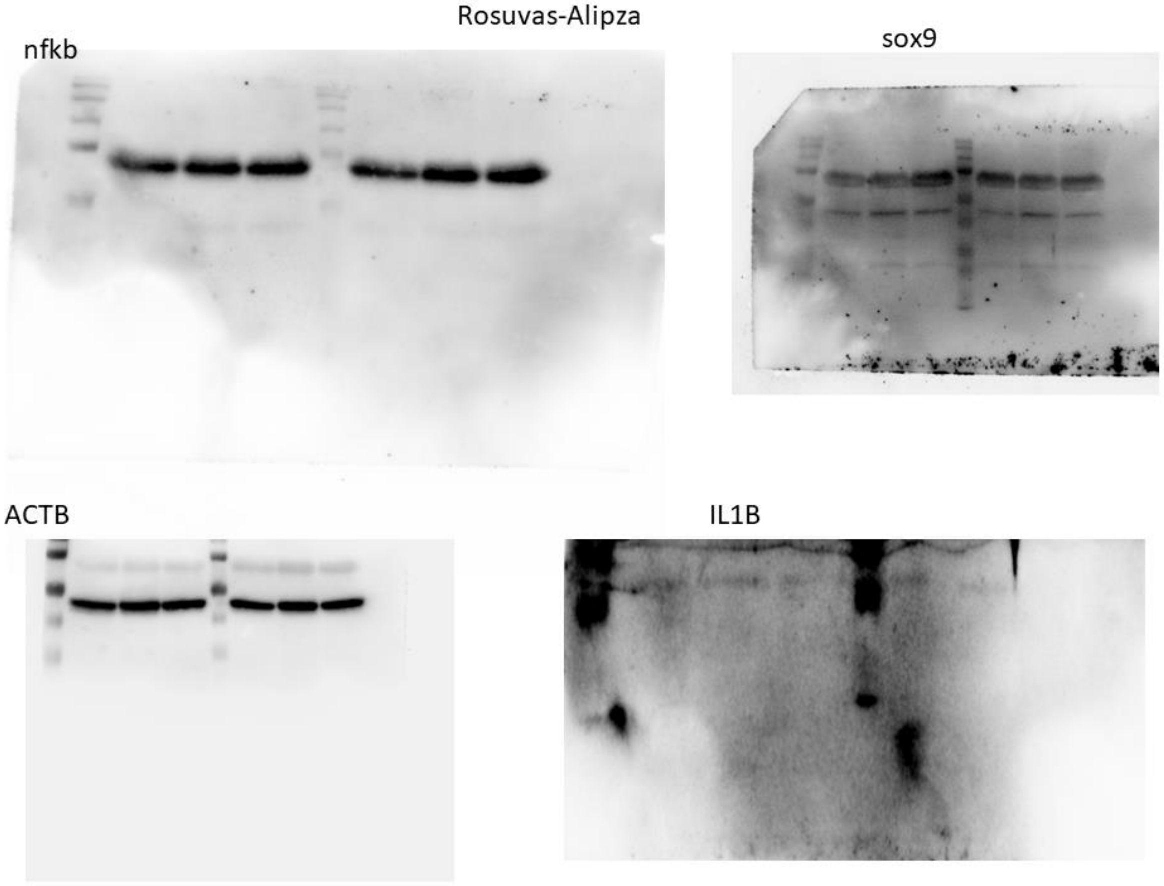

Supplementary Tables; Top 5 Interactions of Pitavastatin and Rosuvastatin with Target Proteins (IL-1β, SOX9, NF-κB)

This tables provides the expanded results of our molecular docking analysis performed using the CB-Dock2 platform. In addition to the highest Vina scores summarized in the main text, this table includes full details for the top 5 results for each drug-protein interaction. The table lists the Vina score, cavity volume (Å³), center coordinates (x, y, z), docking box size, and all amino acid residues involved in the interaction for each hit. This detailed data offers a more in-depth understanding of the potential binding mechanisms of statins with their target proteins.

Table S1. Pitavastatin-IL-1β Docking Analysis

CurPocket ID	Vina score	Cavity volume (Å3)	Center (x, y, z)	Docking size (x, y, z)	Contact residues	Protein-Ligand Docking Visualization
**C2**	-7.4	133	8, -23, 6	22, 22, 22	Chain A: SER21 GLY22 PRO23 TYR24 GLU25 LYS74 ASP75 LYS77 PRO78 THR79 LEU80 GLN81 LEU82 GLU83 SER84 TRP120 SER125 MET130 PRO131 VAL132 PHE133 LEU134 GLY135 GLY136 THR137 ASP142	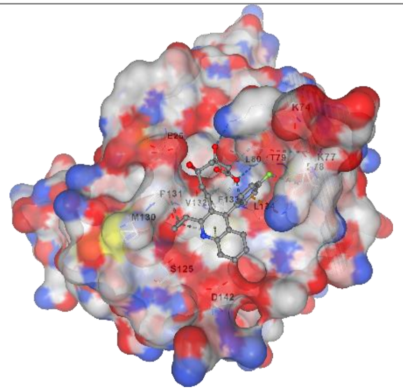
**C1**	-6.9	199	13, -19, -14	22, 22, 22	Chain A: PRO2 VAL3 ARG4 SER5 LEU6 ASN7 PHE42 SER43 MET44 SER45 GLY61 LEU62 LYS63 GLU64 LYS65 ASN66 LEU67 TYR68 VAL85 PRO87 LYS88 ASN89 TYR90 PRO91 LYS92 VAL151 SER152 SER153	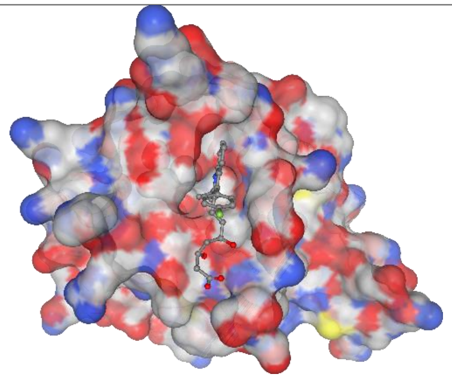
**C5**	-6.6	91	15, -1, -3	22, 22, 22	Chain A: ARG4 LEU6 ARG11 MET44 PHE46 GLU51 SER52 ASN53 ASP54 ILE56 LYS103 GLU105 ASN107 ASN108 LYS109 LEU110 PHE146 THR147 MET148 GLN149 PHE150	

						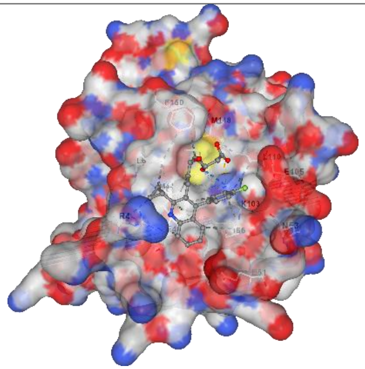
**C4**	-6.5	93	3, -6, -14	22, 22, 22	Chain A: PRO2 VAL3 ARG4 LEU6 MET44 PHE46 VAL47 GLN48 GLY49 GLU50 GLU51 SER52 ASN53 ASP54 LYS55 ILE56 PRO57 PRO91 LYS92 LYS93 LYS94 LYS103 GLU105 LEU110 MET148 PHE150	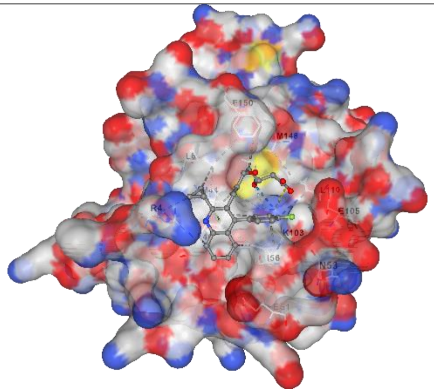

**C3**	-6.1	132	-2, -8,-6	22, 22, 22	Chain A: GLN48 GLY49 GLU50 GLU51 SER52 LYS55 PRO57 MET95 GLU96 LYS97 VAL100 ASN102 GLU113 SER114 ALA115 GLN116 PHE117 PRO118 ASN119	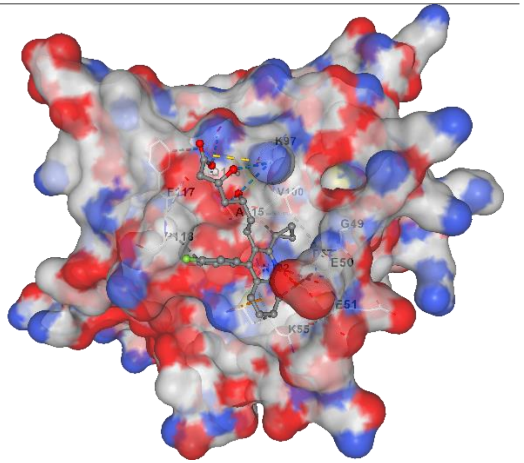

Table S2. Pitavastatin- SOX9 Docking Analysis Table

CurPocket ID	Vina score	Cavity volume (Å3)	Center (x, y, z)	Docking size (x, y, z)	Contact residues	Protein-Ligand Docking Visualization
**C2**	-7.0	347	-4, -19, 16	22, 22, 22	Chain A: ARG107 PRO108 MET109 ASN110 ALA111 PHE112 MET113 TRP143 LYS151 VAL155 ALA158 GLU159 LEU161 ARG162 VAL163 HIS165 LYS166 LYS167 HIS169 PRO170 ASP171 TYR172 LYS173 TYR174	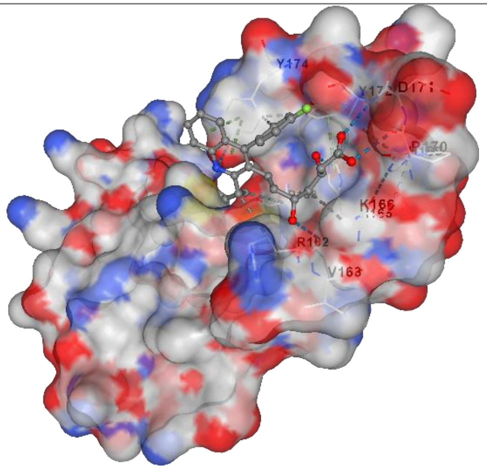

**C5**	-6.2	180	-8, -27, 9	22, 22, 22	Chain A: HIS104 VAL105 LYS106 ARG107 PRO108 MET109 GLN117 GLU157 ARG160 LEU161 GLN164 HIS165 ASP168 HIS169	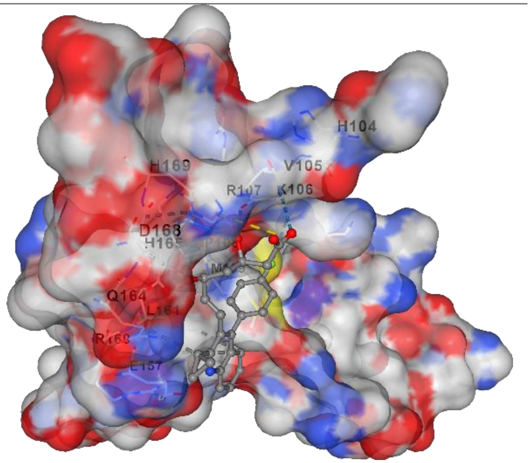
**C1**	-5.3	514	-28, -23, 11	22, 22, 22	Chain A: MET109 PHE112 MET113 ALA116 GLN117 ALA118 ARG120 ARG121 ALA124 ASP125 TYR127 PRO128 HIS129 LEU130 HIS131 ASN132 ALA133 LEU135 SER136	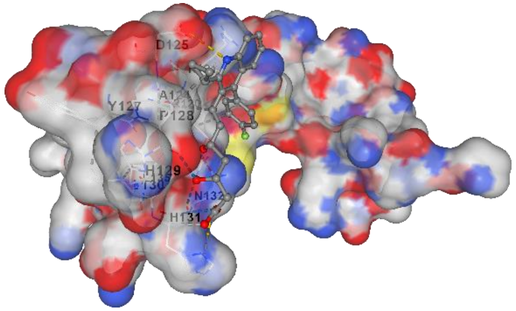

**C3**	-5.3	303	-18, -17, 10	22, 22, 22	Chain A: HIS104 VAL105 LYS106 ARG107 PRO108 MET109 ASN110 PHE112 MET113 VAL114 ALA116 GLN117 ARG120 HIS131 ASN132 ALA133 SER136 LYS137 LEU139 GLY140 TYR172 LYS173 TYR174	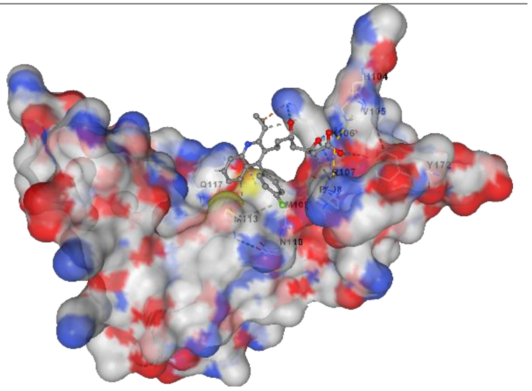
**C4**	-4.1	183	-7, -6, 8	22, 22, 22	Chain A: ARG107 PRO170 ASP171 TYR172 LYS173 TYR174	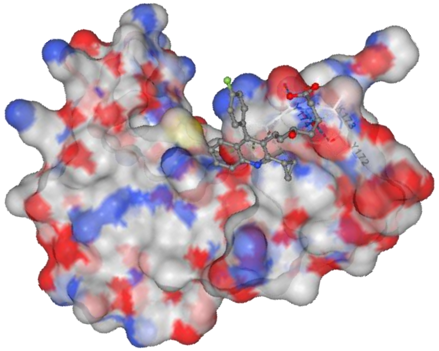

Table S3. Pitavastatin- NF-κB Docking Analysis Table

CurPocket ID	Vina score	Cavity volume (Å3)	Center (x, y, z)	Docking size (x, y, z)	Contact residues	Protein-Ligand Docking Visualization

**C3**	-8.0	904	-11, 53, -11	22, 22, 22	Chain A: ASN200 ARG201 SER205 GLY208 GLY209 ASP210 GLU211 ILE212 PHE213 ARG253 ASP291<br>Chain B: ARG255 ASP257 ARG258<br>Chain F: ASP141 ARG143 GLY144 ASN145 GLU153 ASN180 TYR181 ASN182 GLY183 HIS184 LEU189 ILE192 HIS193 GLU213 CYS215 ARG218 LEU223 ASP226 LEU227	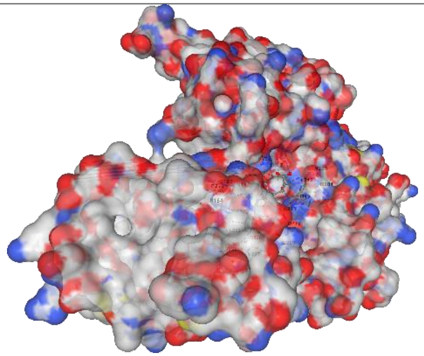
**C4**	-7.8	798	-2, 78, 85	22, 22, 22	Chain C: ASN200 ARG201 SER205 LEU207 GLY208 GLY209 ASP210 GLU211 ILE212 PHE213 ARG253 ASP291<br>Chain D: ARG255 ASP257 ARG258<br>Chain E: ARG143 ASN145 GLU153 ASN180 ASN182 HIS184 LEU189 ILE192 HIS193 GLU213 CYS215 ARG218 LEU223 ASP226 LEU227	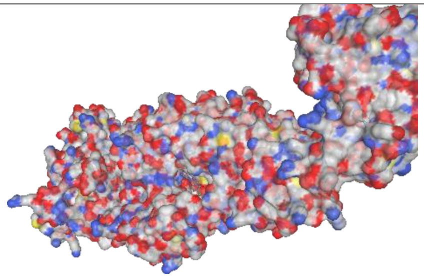
**C2**	-7.6	3002	-7, 51, 11	31, 22, 22	Chain A: LYS28 GLN29 ARG30 GLU49 ARG50 SER51 THR52 LYS79 ASP80 ARG158 HIS181 PRO182 PHE184 LYS218 VAL219 GLN220 LYS221 GLU222 ASP223 ILE224 GLU225 VAL226 SER240 GLN241 ALA242 VAL244 HIS245 ARG246 GLN247 PRO275<br>Chain B: LYS252 VAL254 ASP274	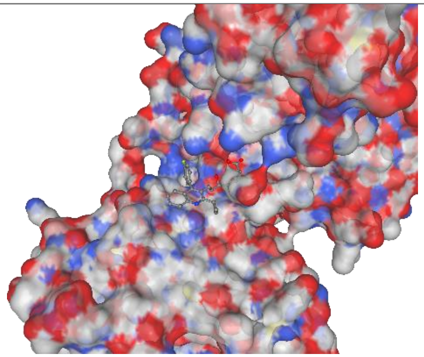

					Chain F: GLN249 GLY250<br>TYR251 THR257 TRP258<br>GLY259 ARG260 MET279<br>LEU280 PRO281 GLU282	
**C1**	-7.3	3597	-3, 79, 106	32, 22, 22	Chain C: GLU25 LYS28<br>GLN29 ARG30 GLU49 ARG50<br>SER51 THR52 ASP53 THR57<br>LYS79 ASP80 ARG158<br>HIS181 PRO182 PHE184<br>LYS218 VAL219 GLN220<br>LYS221 GLU222 ASP223<br>ILE224 GLU225 VAL226<br>PHE239 GLN241 ALA242<br>VAL244 HIS245 ARG246<br>GLN247 VAL248 PRO275<br>Chain D: VAL254 ASP274<br>Chain E: GLN249 TYR251<br>TRP258 GLY259 ARG260<br>LEU280 PRO281 GLU282	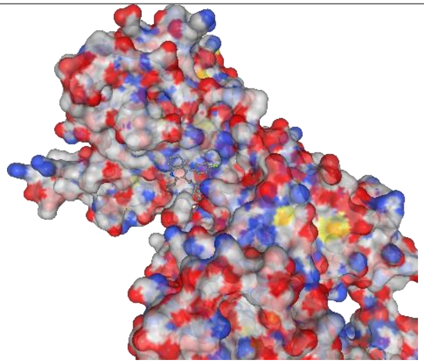
**C5**	-6.2	746	-16, 58, -27	22, 22, 22	Chain B: THR325 LYS326<br>PRO327 ALA328 SER329<br>PRO347 PHE348 LEU349<br>PRO352<br>Chain F: GLN107 GLN111<br>ASP136 GLU138 LEU139<br>ARG140 ASP141 GLY144<br>THR146 HIS149 SER174<br>ILE175 LYS177 ALA178<br>THR179 ASN180 TYR181	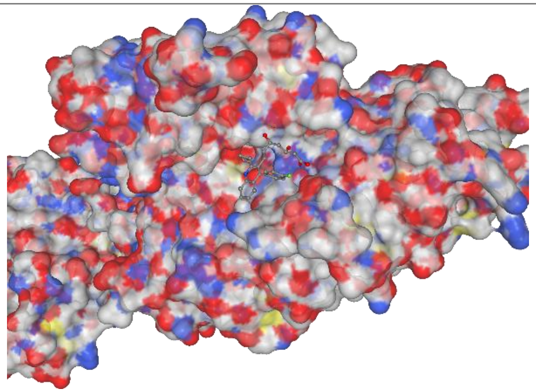

Table S4. Rosuvastatin -IL-1β Docking Analysis Table

CurPocket ID	Vina score	Cavity volume (Å3)	Center (x, y, z)	Docking size (x, y, z)	Contact residues	Protein-Ligand Docking Visualization
**C2**	-7.2	133	8, -23, 6	22, 22, 22	Chain A: SER21 GLY22 PRO23 TYR24 GLU25 LEU26 LEU69 LYS74 LYS77 PRO78 THR79 LEU80 GLN81 LEU82 GLU83 SER84 TRP120 SER123 SER125 MET130 PRO131 VAL132 PHE133 LEU134 GLY135 GLY136 THR137 ASP142	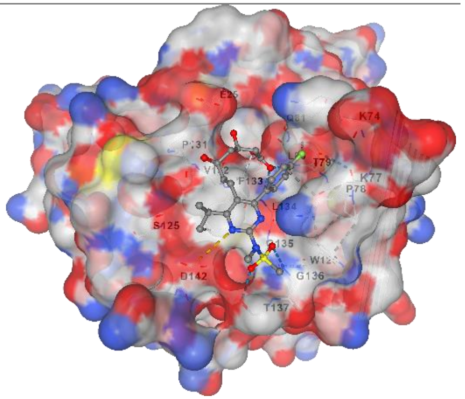
**C1**	-6.4	199	13, -19, -14	22, 22, 22	Chain A: PRO2 VAL3 ARG4 SER5 LEU6 ASN7 SER43 MET44 SER45 GLY61 LEU62 LYS63 GLU64 LYS65 ASN66 LEU67 TYR68 VAL85 ASP86 PRO87 LYS88 ASN89 TYR90 PRO91 LYS92 SER153	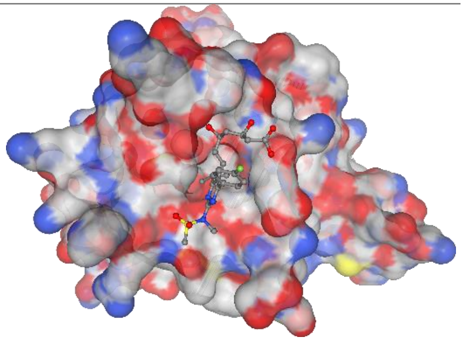

**C5**	-5.9	91	15, -1, -3	22, 22, 22	Chain A: LEU6 ARG11 GLN15 MET44 PHE46 ASN53 ILE56 LYS103 GLU105 ILE106 ASN107 ASN108 LYS109 LEU110 PHE146 THR147 MET148 GLN149 PHE150	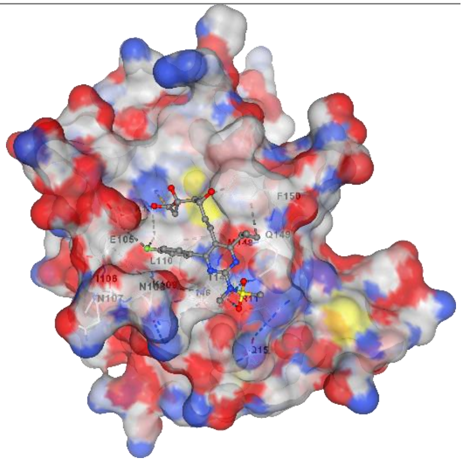
**C3**	-5.7	132	-2, -8, -6	22, 22, 22	Chain A: GLN48 GLY49 GLU50 GLU51 SER52 LYS55 PRO57 LYS93 LYS94 MET95 GLU96 LYS97 VAL100 ASN102 GLU113 SER114 ALA115 GLN116 PHE117 PRO118 ASN119	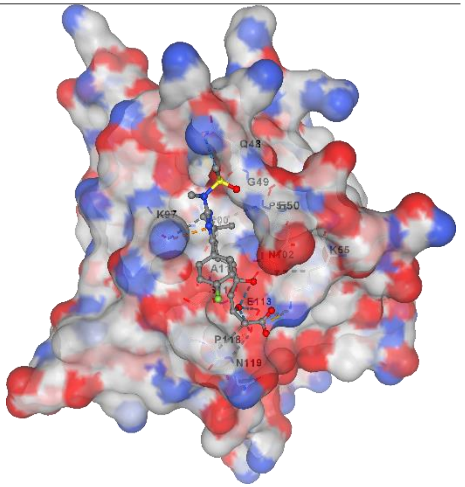

**C4**	-5.7	93	3, -6, -14	22, 22, 22	Chain A: PRO2 VAL3 ARG4 LEU6 MET44 SER45 PHE46 VAL47 GLN48 GLU51 SER52 ASN53 ASP54 LYS55 ILE56 PRO57 VAL58 LYS93 LYS103 GLU105 MET148 PHE150	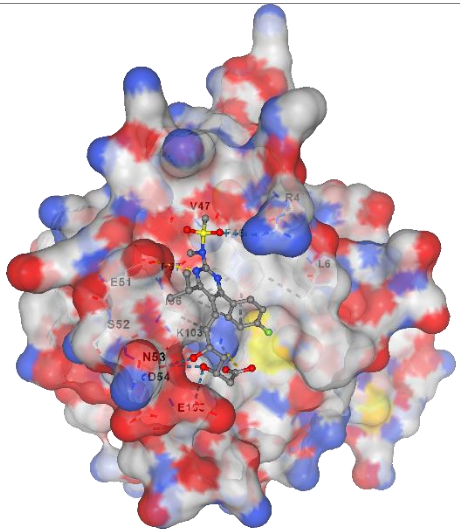

Table S5. Rosuvastatin -SOX9 Docking Analysis Table

CurPocket ID	Vina score	Cavity volume (Å3)	Center (x, y, z)	Docking size (x, y, z)	Contact residues	Protein-Ligand Docking Visualization
**C2**	-5.8	347	-4, -19, 16	22, 22, 22	Chain A: ARG107 PRO108 MET109 ASN110 ALA111 PHE112 MET113 TRP143 LYS151 VAL155 GLU156 ALA158 GLU159 ARG162 VAL163 HIS165 LYS166 LYS167 HIS169 PRO170 ASP171 TYR172 LYS173 TYR174	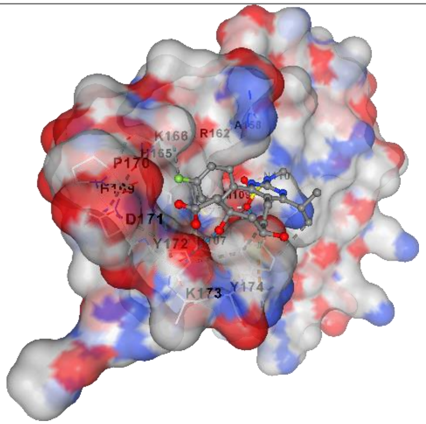
**C5**	-5.5	180	-8, -27, 9	22, 22, 22	Chain A: HIS104 VAL105 LYS106 ARG107 PRO108 MET109 VAL114 GLN117 GLU157 ARG160 LEU161 ARG162 GLN164 HIS165 ASP168 HIS169	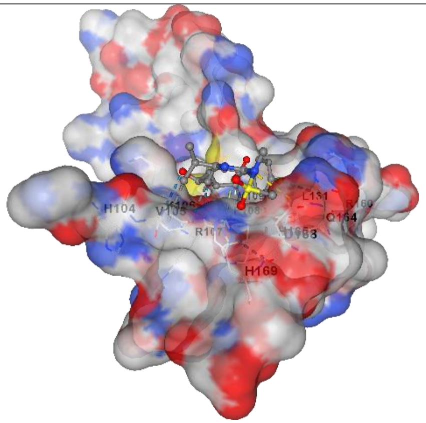

**C1**	-5.2	514	-28, -23, 11	22, 22, 22	Chain A: MET109 PHE112 MET113 ALA116 GLN117 ALA118 ARG120 ARG121 ALA124 ASP125 GLN126 TYR127 PRO128 HIS129 LEU130 HIS131 ASN132 ALA133 LEU135 SER136	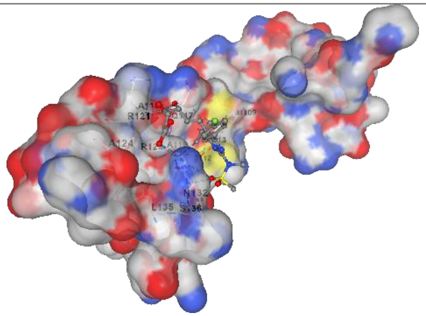
**C3**	-5.2	303	-18, -17, 10	22, 22, 22	Chain A: HIS104 VAL105 LYS106 ARG107 PRO108 MET109 ASN110 PHE112 MET113 VAL114 TRP115 ALA116 GLN117 ARG120 HIS131 ASN132 ALA133 SER136 TYR172 LYS173 TYR174	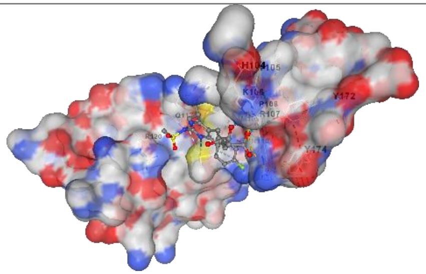
**C4**	-3.2	183	-7, -6, 8	22, 22, 22	Chain A: ARG107 ARG162 ASP171 TYR172 LYS173 TYR174	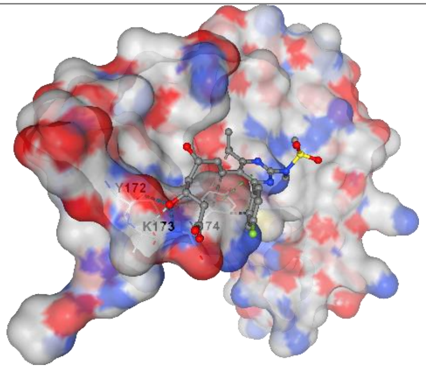

Table 6S. Rosuvastatin - NF-κB Docking Analysis Table

CurPocket ID	Vina score	Cavity volume (Å3)	Center (x, y, z)	Docking size (x, y, z)	Contact residues	Protein-Ligand Docking Visualization
**C1**	-7.9	3597	-3, 79, 106	32, 22, 22	Chain C: LYS28 ARG30 GLY31 GLU49 ARG50 SER51 THR52 VAL219 GLN220 LYS221 GLU222 ASP223 ILE224 GLU225 SER238 PHE239 SER240 GLN241 ALA242 VAL244 HIS245 ARG246 GLN247 PRO275 SER276 Chain D: ASN250 LYS252 VAL254 ASP274 Chain E: TYR248 GLN249 GLY250 TYR251 THR257 TRP258 GLY259 ARG260 PRO261 SER262 GLN266 MET279 LEU280 PRO281 GLU282	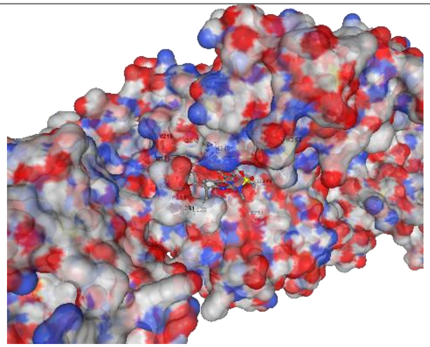
**C3**	-7.3	904	-11, 53, -11	22, 22, 22	Chain A: ASN200 ARG201 GLY208 GLY209 ASP210 GLU211 ILE212 PHE213 ARG253 Chain B: ARG255 MET256 ASP257 ARG258 Chain F: ARG143 ASN145 LEU150 GLU153 ASN180 TYR181 ASN182 GLY183 HIS184 LEU189 ILE192 HIS193 GLU213 CYS215 ASN216 ARG218 LEU223 ASP226 LEU227	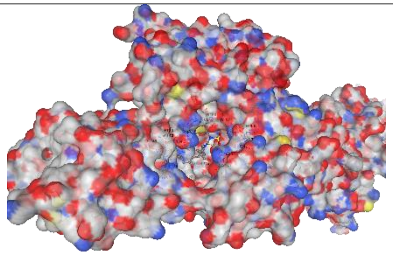

**C2**	-7.0	3002	-7, 51, 11	31, 22, 22	Chain A: LYS28 GLN29 ARG30 GLY31 PRO47 GLU49 ARG50 SER51 THR52 ASP53 THR54 LYS79 ASP80 ARG158 HIS181 PRO182 PHE184 LYS218 VAL219 GLN220 LYS221 GLU222 ASP223 ILE224 GLU225 VAL226 ARG236 GLY237 SER238 PHE239 SER240 GLN241 ALA242 VAL244 HIS245 ARG246 GLN247 ARG274 PRO275 SER276<br>Chain F: THR257 TRP258 GLY259 ARG260 PRO261 SER262 THR263 GLN266 MET279 LEU280 PRO281 GLU282	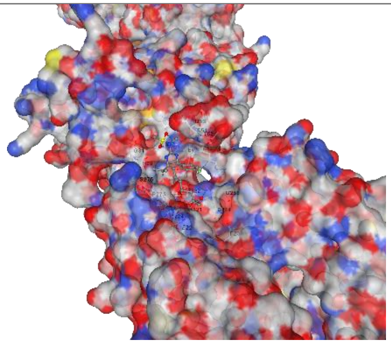
**C4**	-7.0	798	-2, 78, 85	22, 22, 22	Chain C: ASN200 ARG201 SER203 GLY204 SER205 LEU207 GLY208 GLY209 ASP210 GLU211 ILE212 PHE213 ARG253<br>Chain D: ARG255 MET256 ASP257 ARG258<br>Chain E: ARG143 ASN145 GLU153 ASN180 TYR181 ASN182 GLY183 HIS184 LEU189 ILE192 HIS193 GLU213 PRO214 CYS215 LEU223 ASP226 LEU227	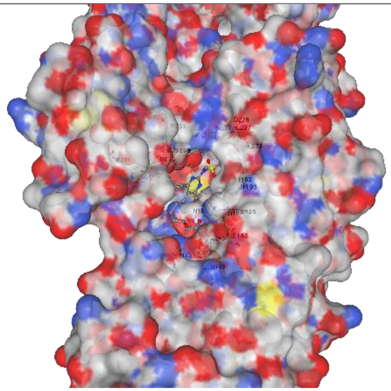

**C5**	-5.5	746	-16, 58, -27	22, 22, 22	Chain B: ILE322 ASN323 ILE324 THR325 LYS326 PRO327 ALA328 SER329 LEU349 TYR351 PRO352 GLU353 ILE354 LYS355 Chain F: GLY74 ASN105 GLN107 ASN108 ASN109 GLN111 ASP136 GLU138 LEU139 ARG140 ASP141 HIS173 SER174 ILE175 LYS177 ALA178 THR179 ASN180 TYR181	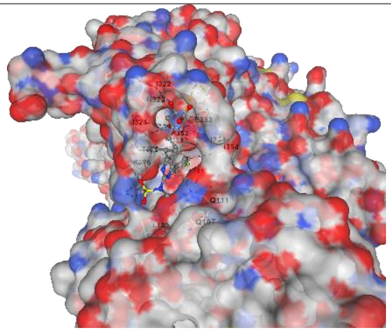
